# Osteoclasts differential-related prognostic biomarker for osteosarcoma based on single cell, bulk cell and gene expression datasets

**DOI:** 10.1186/s12885-022-09380-z

**Published:** 2022-03-17

**Authors:** Haiyu Shao, Meng Ge, Jun Zhang, Tingxiao Zhao, Shuijun Zhang

**Affiliations:** 1 Department of Orthopaedics, Zhejiang Provincial People’s Hospital, Affiliated People’s Hospital, Hangzhou Medical College, Shangtang Road 158#, Hangzhou, 310014 Zhejiang China; 2 Department of Orthopaedics, Bengbu Medical College, Bengbu, Anhui China

**Keywords:** Osteosarcoma, Osteoclasts, Differentiation, Prognostic, scRNA-seq

## Abstract

Osteosarcoma (OS) is one of the most common primary bone malignant tumors. Osteoclasts have been shown to have a valuable role in OS. In the present study, we analyzed the differentiation states of osteoclasts in OS and their prognostic significance based on integrated scRNA-seq and bulk RNA-seq data. Osteoclasts in distinct differentiation states were characterized, and 661 osteoclasts differentiation-related genes (ODRGs) were obtained. ORDGs in distinct differentiation states were enriched in distinct functions and pathways. TPM1, S100A13, LOXL1, PSMD10, ST3GAL4, PEF1, SERPINE2, TUBB, FAM207A, TUBA1A, and DCN were identified as the significant survival-predicting ODRGs. We successfully developed a risk score model based on these survival-predicting ODRGs. In addition, we generated a nomogram applicable for clinical with both ODRGs signatures and clinicopathological parameters, and validated in OS cohorts to predict OS patient outcome. This study proposed and verified the important roles of osteoclasts differentiation in the prognosis of patients with OS, suggesting promising therapeutic targets for OS.

**Supplementary Information:**

The online version contains supplementary material available at 10.1186/s12885-022-09380-z.

## Introduction

As one of the most common primary bone malignant tumors [1], the incidence of osteosarcoma (OS) in the general population is 2–3 million/year. However, the incidence of OS is higher among adolescents, with a maximum incidence of 8–11 million per year in adolescents aged 15–19 years [2]. The typical symptoms of OS are local pain, local swelling, and limited joint movement. Due to advances in the treatment of OS in the preliminary stage, the 5-year survival rate or long-term survival rate for patients with OS has been greatly improved [3–5]. Unsatisfactorily, this trend of improvement seems to have stalled and entered a bottleneck period in the past 20 years. Although there have been some reports on prognostic predictors for patients with OS, such as CBX3 [6], LSINCT5 [7], MCT4 [8], and serum LDH [9]. However, the current predictive models are far from satisfactory.

The osteoclasts have a unique role in bone resorption and play a key role in skeletal pathology with evident bone destruction [10]. Osteoclasts are coupled with new bone formation synthesized by osteoblasts [11]. During the development of OS, osteoblasts or bone-forming cells form or secrete osteoid [12]. Based on the above, conventional OS cells are defined as osteoblast cell lines, which play an inducible role in osteoclastogenesis by secreting osteoclast-inducing factors [10]. Several studies have shown that osteoclasts have a valuable role in OS [13–15]. Moreover, osteoclast-targeted therapy may be a better option for OS compared to other bone tumors. Bisphosphonates control osteoclasts differentiation, bone resorption activity and other functions, and have led to advances in new therapies against bone tumors, such as OS [16]. However, it is unclear whether osteoclasts in different differentiated states and osteoclasts differentiation-related genes play a role in predicting patient survival in OS.

Therefore, in this study, we identified two osteoclasts’ subsets with different differentiation states using trajectory analysis of scRNA-seq data and identified significant osteoclasts differentiation-related genes (ODRGs). Next, we investigated these ODRGs and their biological functions. Then, significant prognostic ODRGs were obtained and the prognostic risk model was established. Finally, a clinically applicable prognostic nomogram for OS patients was developed by combining prognostic ODRGs with other clinicopathological variables. Our findings suggested that ODRGs are significant in the prognostic process and might serve as a promising target for OS treatment.

## Materials and methods

### Data collection

In this study, we analyzed the scRNA-seq and bulk RNA-seq data of human OS samples. We obtained 11 OS samples (GSE152048, **Table ****1**) with scRNA-seq data based on the 10X Genomics platform from GEO database (http://www.ncbi.nlm.nih.gov/geo/). We obtained the bulk RNA-seq and clinical data of OS samples from TARGET database (https://ocg.cancer.gov/programs/target/data-matrix), containing 84 samples with survival data. Additionally, OS microarray expression data in GSE39055 from GEO database was obtained for prognostic risk model validation.

**Table 1:** Details of the osteosarcoma samples used in this study

Sample	Type	Location	Size(cm)
BC2	Primary	Femur	5.5*5*3
BC3	Primary	Tibia	8*6*6
BC5	Primary	Femur	8*7.5*6
BC6	Primary	Ulna	7*7*4
BC10	Metastasis(Lung)	Femur	3.5*3*2
BC16	Primary	Tibia	6*4*2.5

### Processing of the scRNA-seq data

Five primary tumor samples of conventional pathological type and 1 lung metastasis sample in the GSE152048 dataset were used for analysis. The scRNA-seq data was analyzed statistically by seurat package [17]. First of all, cells with the following conditions were excluded: 1) cells with < 300 total detected genes; 2) cells with ≥ 10% of mitochondria-expressed genes; and 3) genes detected in < 5 cells. Next, the linear regression model was applied to normalize gene expression in the remaining cells. The batch effect of 5 primary tumor (BC2, BC3, BC5, BC6, and BC16) was eliminated using the IntegrateData of Seurat package, and the 5 samples were integrated. The identification of significantly available dimensions was conducted using PCA with the criteria of *P* < 0.05. Afterwards, 30 initial principal components (PCs) were dimensionality reduced using the t-distributed stochastic neighbor embedding (tSNE) algorithm, and all cells were conducted analysis of cluster classification. Cell clusters were annotated according to the marker genes obtained from the literatures and the CellMarker Database (Supplementary Table 1).

### Trajectory analysis and osteoclasts differential related genes (ODRGs) identification

Monocle 2 algorithm was used to conduct single-cell pseudotime trajectories of the osteoclasts. Single cells were arranged in a trajectory with branch points. Cells of different branches were thought to have different characteristics of cell differentiation, likewise the cells of the same branch were in the same state of differentiation. Hereafter, differential expressed genes between branches were analyzed, and the differential expressed genes were defined as marker genes. ODRGs are osteoclasts cells marker genes located in different branches.

### GO and KEGG enrichment analysis of branch-dependent ODRGs

GO and KEGG (https://www.kegg.jp/kegg/kegg1.html) enrichment analysis of ODRGs on different branches was conducted using the Clusterprofiler v3.16.1 [18]. The results were presented as bubble plots.

### Development and validation of ODRG-based prognostic risk score model

First, in the TARGET OS cohort, the associations between ODRGs levels and patient survival were assessed using the univariate Cox regression analysis (*P* < 0.05). TARGET OS cohort was first split into training and testing datasets, with 58 samples in the training data (70%) and 26 samples (30%) in the testing data. Prognosis-related genes were first identified using criteria with *P* < 0.05, followed by further screening by Cox-LASSO regression analysis with R package glmnet. Finally, the prognostic signature of OS based on ODRGs expressions and their relevant coefficients result from above analysis were constructed. The formula is as follows: $\mathrm{Risk score}= {\sum }_{1}^{N}({coef}_{i} \times {expr}_{i})$, in which “expr” refers to the corresponding gene expression, and “coef” refers to the regression coefficient calculated by the LASSO analysis. The samples were split into high-risk and low-risk groups based on the median of Risk score. The overall survival difference between the low-risk group and the high-risk group was assessed by Kaplan–Meier survival assay with log-rank test in the TARGET testing dataset and the entire TARGET cohort. Receiver operating characteristic (ROC) curve analysis was applied for evaluating the sensitivity and specificity of ODRGs signature. Moreover, univariate and multivariate Cox regression analysis were performed to determine whether the prognostic value of ODRGs signature was influenced by other clinical features.

### GSEA analysis of high-risk and Low-risk groups in TARGET OS cohort

To explore the differences in gene function in different risk groups, the samples of different risk groups were analyzed by KEGG enrichment analysis using GSEA.

### Verification of signatures based on ODRGs

The data of GSE39055 was used to verify the ODRGs signatures. According to the established prognostic risk score model, the risk score of each patient was calculated. Likewise, the patients were divided into a high-risk group and a low-risk group based on the median value. The overall survival difference of different groups was evaluated by Kaplan–Meier survival assay with log-rank test. Moreover, the receiver operating characteristic (ROC) curve was plotted and the area under the curve (AUC) was calculated.

### Construction and evaluation of nomograms

All the identified independent prognostic parameters were applied to construct a prognostic nomogram for the 1-, 3-, and 5-year survival rates prediction of OS patients after univariate and multivariate Cox regression analyses. The calibration plots at 3-, and 5- years graphically assessed the discriminative ability of the nomogram.

### Statistical Analysis

Kaplan–Meier statistics and log-rank tests were used for survival analysis. R software version 3.5.2 and corresponding packages were applied for statistical analysis and graphical calculations. *P* < 0.05 was considered to be statistically significant.

## Results

### Identification of clusters in human OS cells using scRNA-seq data reveals high cell heterogeneity

**Fig. 1:**
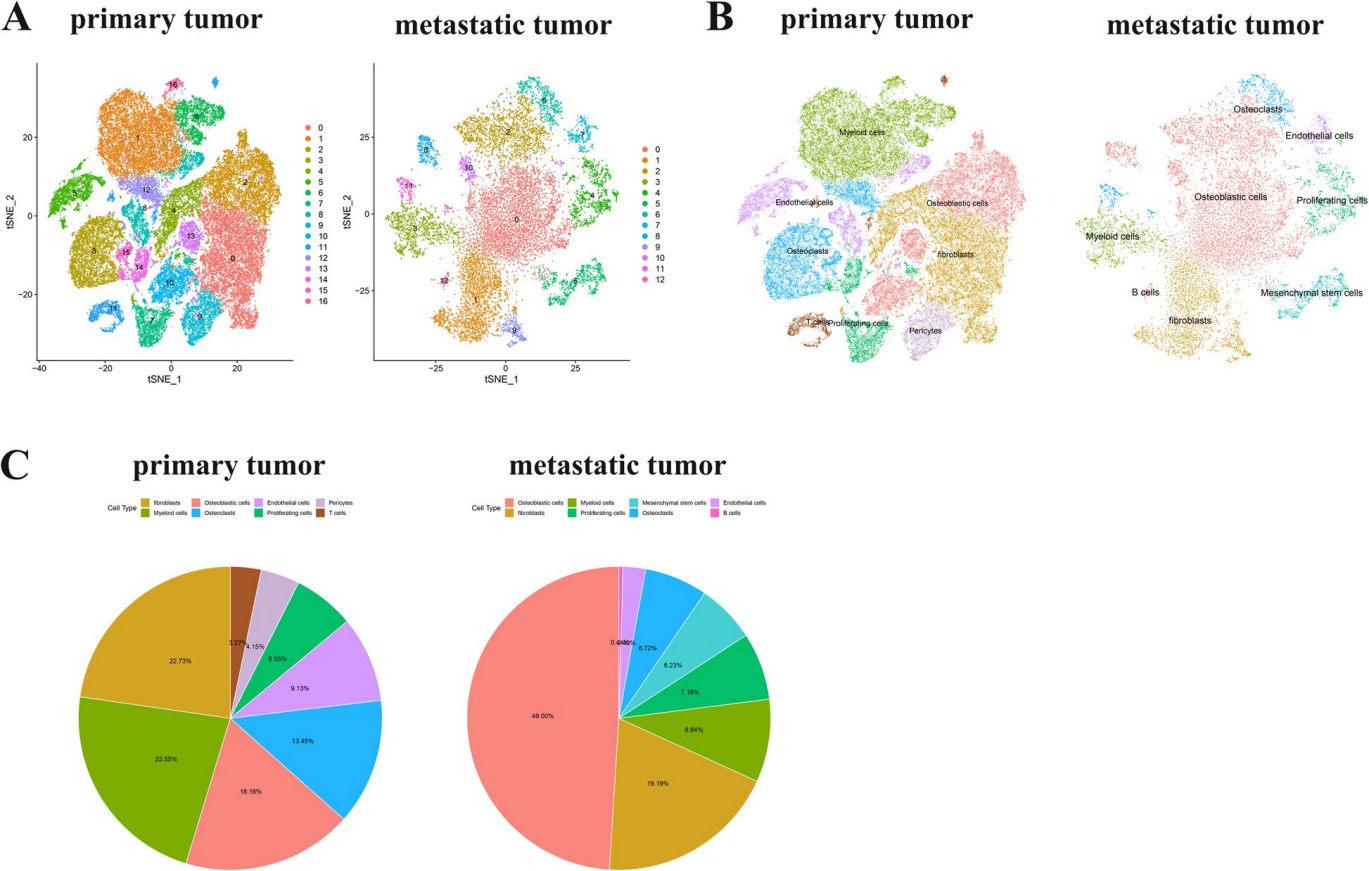
**A** The tSNE algorithm for dimensionality reduction with the 30 PCs, and separate clusters were classified in primary and metastasis tumor cells. **B** Separate clusters of cells in primary and metastasis tumor cells were annotated by literatures and CellMarker according to the composition of the marker genes. **C** Proportion of cell types in primary and metastatic tumor cells

After quality control and batch effect-correction, OS scRNA-seq data was normalized. 60,204 genes and 21,676 cells from OS primary tumor, 19,219 genes and 15,662 cells from OS metastasis tumor were included in the analysis. At the beginning, the determination of available dimensions and the screening of related genes were performed using the principal component analysis (PCA). Here, we selected 30 initial principal components (PCs, *P* < 0.05), followed by t-distributed stochastic neighbor embedding (tSNE) algorithm, which was applied for dimensionality reduction of 30 initial PCs. Then, cluster classification analysis was performed on all cells. 17 separate clusters were found in primary tumor cells, and 13 separate clusters were identified in metastasis tumor cells (Fig. 1A). Afterward, these clusters were annotated by cell types based on the expression of marker genes in clusters according to the CellMarker database and literatures (Fig. 1B, C). The cells of primary tumor cells were annotated as fibroblasts, myeloid cells, osteoblastic cells, osteoclasts, endothelial cells, proliferating cells, pericytes, and T cells. And the cells of metastasis tumor were annotated as osteoblastic cells, fibroblasts, myeloid cells, proliferating cells, mesenchymal stem cells, osteoclasts, endothelial cells, and B cells.

### Osteoclasts can be divided into two subsets with distinct differentiation patterns

**Fig. 2:**
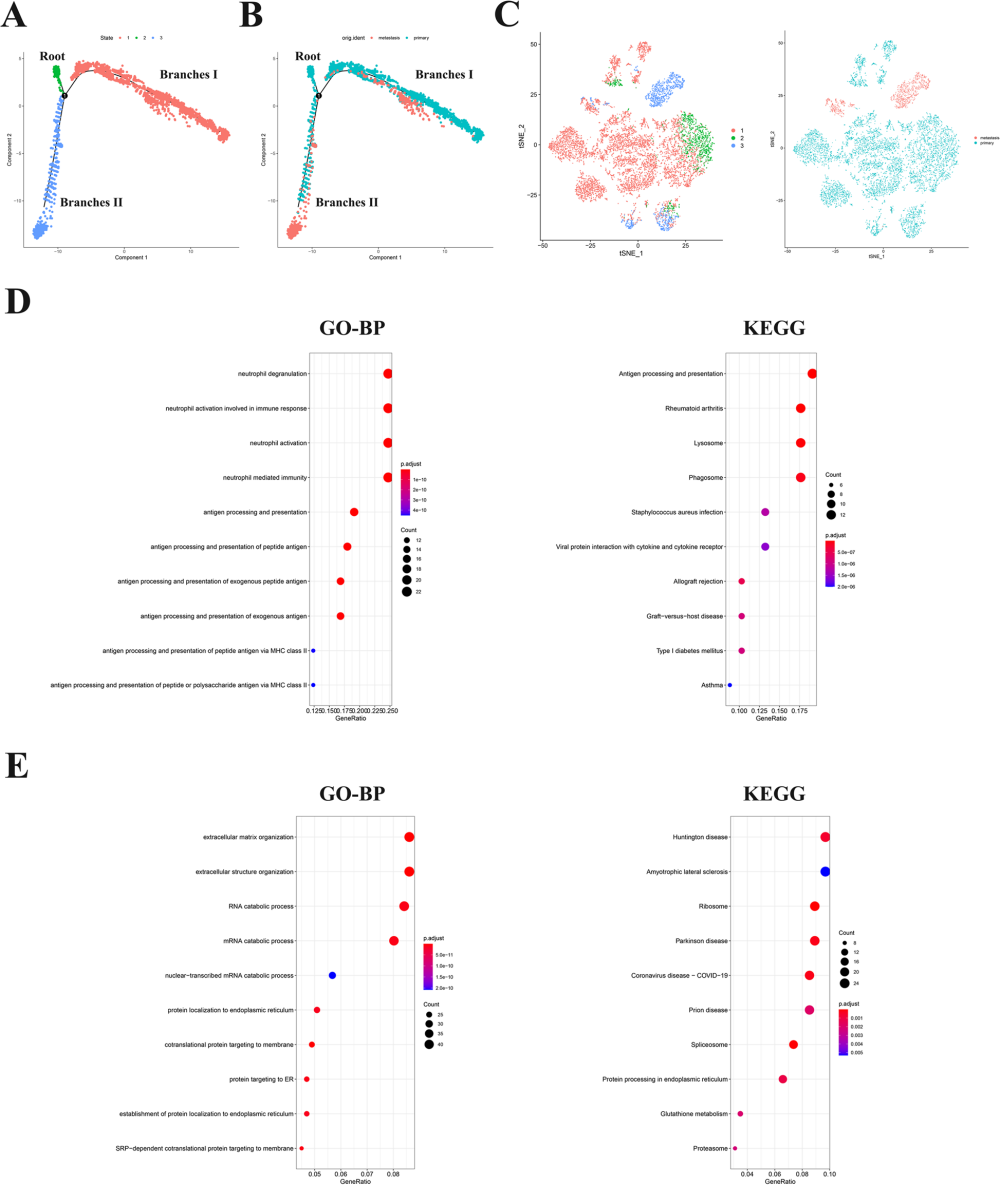
**A-B** Trajectory analysis revealed osteoclasts from primary and metastatic tumor with distinct differentiation patterns. **C** The t-SNE algorithm was conducted based on available significant components. **D, E** GO and KEGG enrichment analysis of ODRGs in branch I and II were performed

All osteoclasts cells from OS were projected onto one root and branches I and II by trajectory analysis (Fig. 2A, B). The results demonstrated that osteoclasts in the primary tumor were mainly located in the branches I, whereas osteoclasts in metastatic tumor were mostly located in the branches II. The root was distributed by osteoclasts from primary tumor. In conventional data interpretation, cells of the same branch were generally defined as being in the same differentiation state, while cells of different branches have different characteristics of cell differentiation. Therefore, these osteoclasts marker genes located in branches I or II were regarded as osteoclasts differentiation related genes (ODRGs). 104 marker genes in branches I and 557 marker genes in branches II were identified as ODRGs using differential expression analysis (Fig. 2C, Supplementary Fig. 1). The molecular functions and pathways of ODRGs in different branches were conducted by GO and KEGG enrichment analysis. Figure 2D, E confirmed that ODRGs in branch I were mainly enriched in neutrophil degranulation, neutrophil activation involved in immune response and other immune-related pathways, ODRGs in branch II were mainly enriched in the extracellular matrix organization, extracellular structure organization and other pathways.

### Prediction of prognostic ODRGs biomarker

**Fig. 3:**
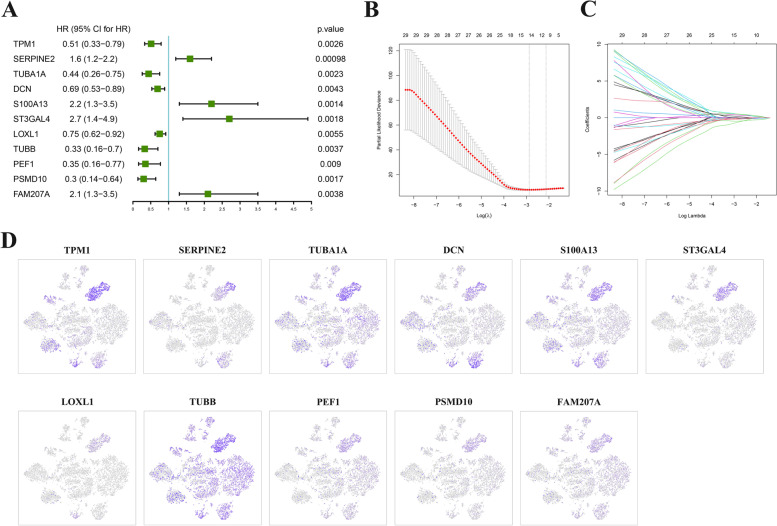
**A** Forest plots of 11 significantly survival-related ODRGs. **B** Ten-fold cross-validation for tuning parameter selection in the LASSO model. **C** LASSO coefficient profiles of the 11 significantly survival-related ODRGs. **D** The expression of the 11 significant survival-predicting ODRGs in osteoclasts

We next investigated associations between 661 ODRGs and overall survival in the TARGET dataset by univariate analysis (Supplementary Table 2). TARGET OS cohort was first split into training and testing datasets, with 58 samples in the training data (70%) and 26 samples (30%) in the testing data. According to the selection criteria with a *P* value < 0.05, 85 prognostic associated ODRGs were selected out (Supplementary Table 2). Cox-LASSO regression analysis was then performed in the TARGET training dataset, and 11 significant survival-predicting ODRGs were identified (Fig.3A-C). The results of expression levels of the 11 significant survival-predicting ODRGs in osteoclasts demonstrated that they were highly expressed mainly in metastatic tumor cells (Fig.3D).

### Prognostic risk model construction

**Fig. 4:**
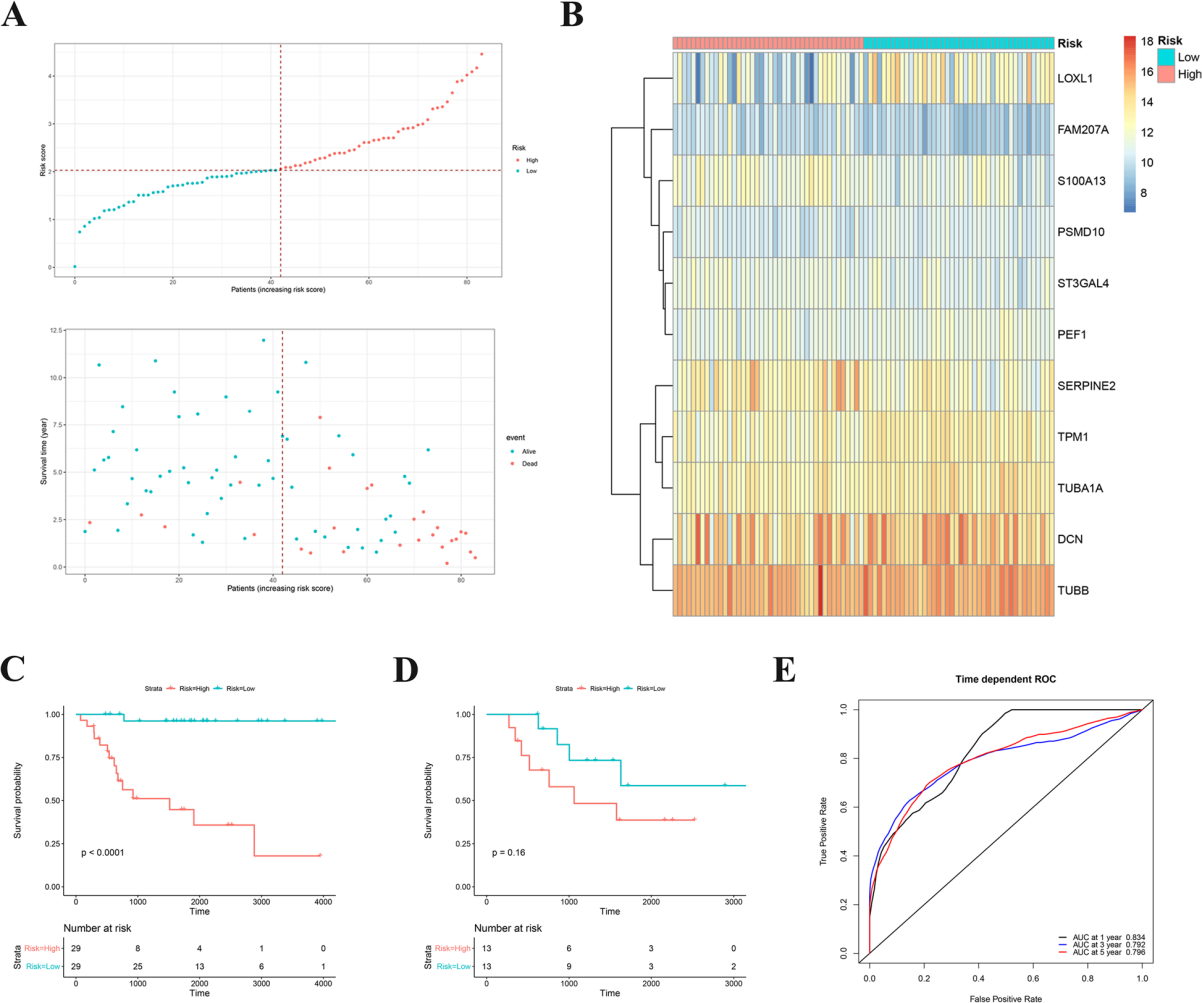
**A** Risk score analysis of the significantly survival-related ODRGs signatures in the TARGET OS cohorts were calculated. The upper figure showed that risk score curves of the significantly survival-related ODRGs signatures. The bottom figure showed that patient survival status and time distributed by the risk score. **B** Heatmap of 11 significantly survival-related ODRGs. **C-D** Kaplan–Meier analysis of different risk group in training data and testing data. **E** Prediction the 1-, 3- and 5-year OS rates the based on ODRGs signature in TARGET OS cohorts was performed by time-dependent ROC curve analysis

Based on 11 survival-related ODRGs, the prognostic risk model was constructed in TARGET training dataset. Its calculation is as follows: risk score = -0.3072 × (TPM1 expression level) + 0.2282 × (SERPINE2 expression level) + -0.0369 × (TUBA1A expression level) + -0.0618 × (DCN expression level) + 0.2319 × (S100A13 expression level) + 0.1904 × (ST3GAL4 expression level) + -0.113 × (LOXL1 expression level) + -0.0527 × (TUBB expression level) + -0.0465 × (PEF1 expression level) + -0.0549 × (PSMD10 expression level) + 0.3118 × (FAM207A expression level). According the median cutoff value of the risk scores, OS patients were split into low risk group and high risk group (Fig. 4A, B). First, Kaplan–Meier analysis of high or low risk groups was conducted on training data and testing data in TARGET dataset, respectively. It was found that the high-risk group in training data was obviously associated with shorter survival time (*P* < 0.0001, Fig. 4C). While there was no significant correlation in testing data, which may be related to the lack of a sufficient number of samples (*P* = 0.16, Fig. 4D). To further verify whether the prognostic risk score model has a good sensitivity and specificity, we conducted receiver operating characteristic (ROC) curve analysis of TARGET OS cohorts. As shown in the results of Fig. 4E, ODRGs signature served as an excellent predictor of 1-, 3- and 5-year OS rates, with respective area under the curve (AUC) values of 0.834, 0.792 and 0.796, respectively.

**Fig. 5:**
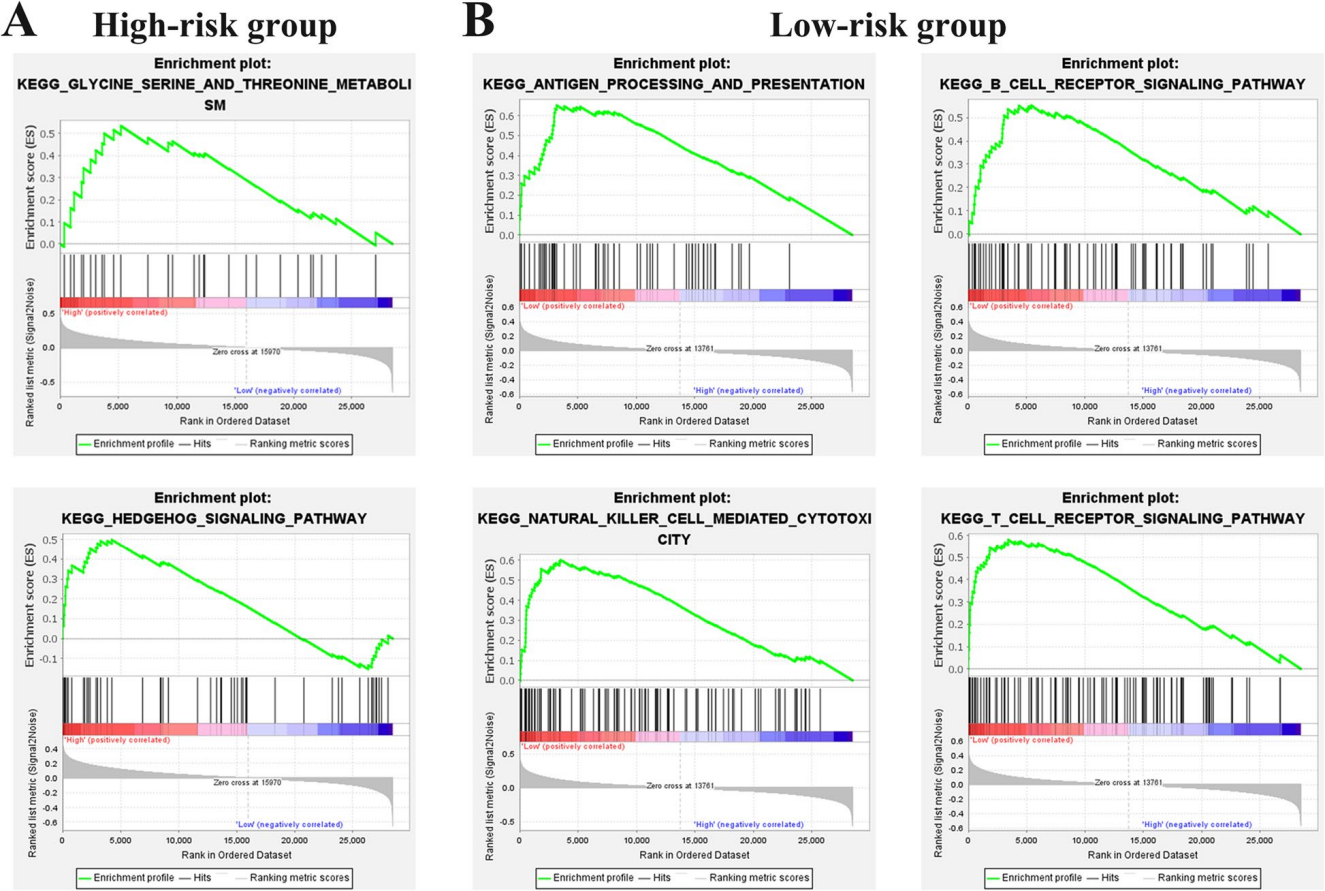
**A, B** GSEA analysis showed the pathways enriched in high and low risk groups

Moreover, the significant pathways in different risk groups in TARGET OS cohorts were investigated using the GSEA analysis. 2 KEGG terms and 4 KEGG terms were enriched in the high and low risk groups, respectively (Fig. 5A, B).

**Fig. 6:**
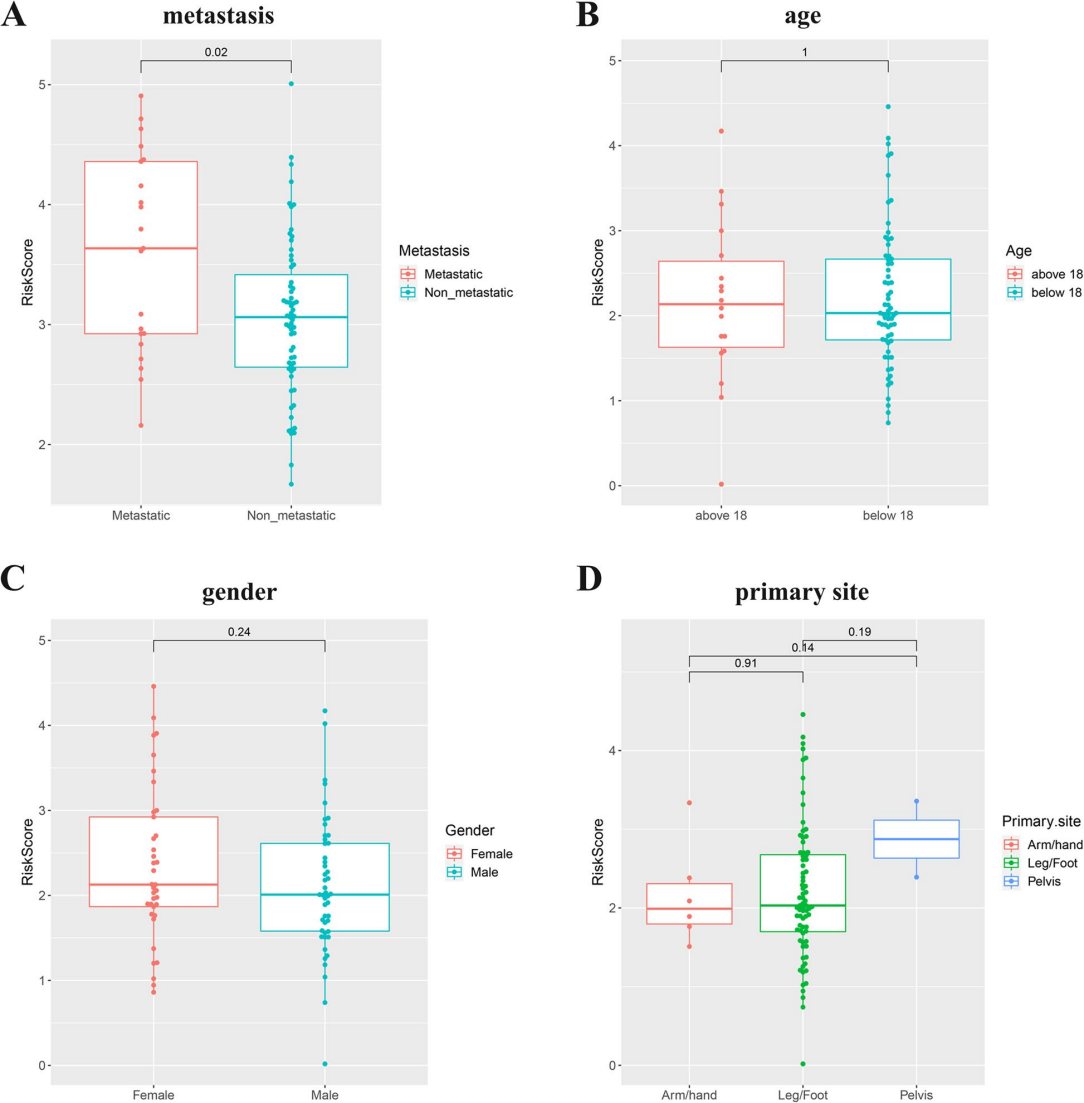
**A-D** Correlation analysis of the risk score and metastasis, age, gender or primary site in the TARGET OS cohorts

Additionally, to evaluate the associations between risk score and clinical characteristics in TARGET OS cohorts, correlation analysis was performed. Correlation analysis demonstrated that risk score was remarkably correlated to metastasis (Fig. 6A). There was no significant correlation with age, gender or primary site (Fig. 6B-D).

### Validation of the ODRGs-based prognostic risk score model

**Fig. 7:**
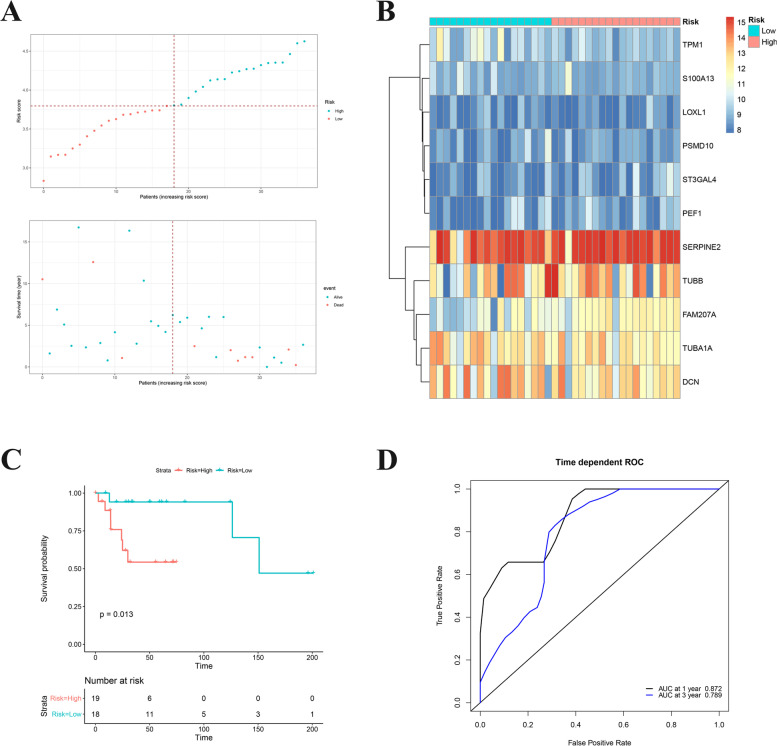
**A** Risk score analysis of the significantly survival-related ODRGs signatures in the TARGET OS cohorts were calculated. The upper figure showed that risk score curves of the significantly survival-related ODRGs signatures. The bottom figure showed that patient survival status and time distributed by the risk score. **B** Heatmap of 11 significantly survival-related ODRGs. **C** Kaplan–Meier analysis of different risk group in GSE39055 cohort. **D** The 1- and 3-year OS rates based on ODRGs signature in GSE39055 cohort were predicted by time-dependent ROC curve analysis

Next, GSE39055 cohort was used to validate the ODRGs-based prognostic risk score model. First, OS samples in GSE39055 cohort were split into high-risk or low-risk groups according to the above method (Fig. 7A-B). The results of the survival analysis were consistent with the results in Fig. 4C, where the overall survival rate in the high-risk group was notably lower than in the low-risk group (*P* = 0.013, Fig. 7C). The ROC analysis also confirmed the sensitivity and specificity of this model in the GSE39055 validation data (Fig. 7D). The above findings uncovered that the prognostic risk score model based on these ODRGs could act as a prognostic predictor for OS patients.

### Development and validation of the clinically applicable prognostic nomogram with the risk score and clinicopathological parameters

**Fig. 8:**
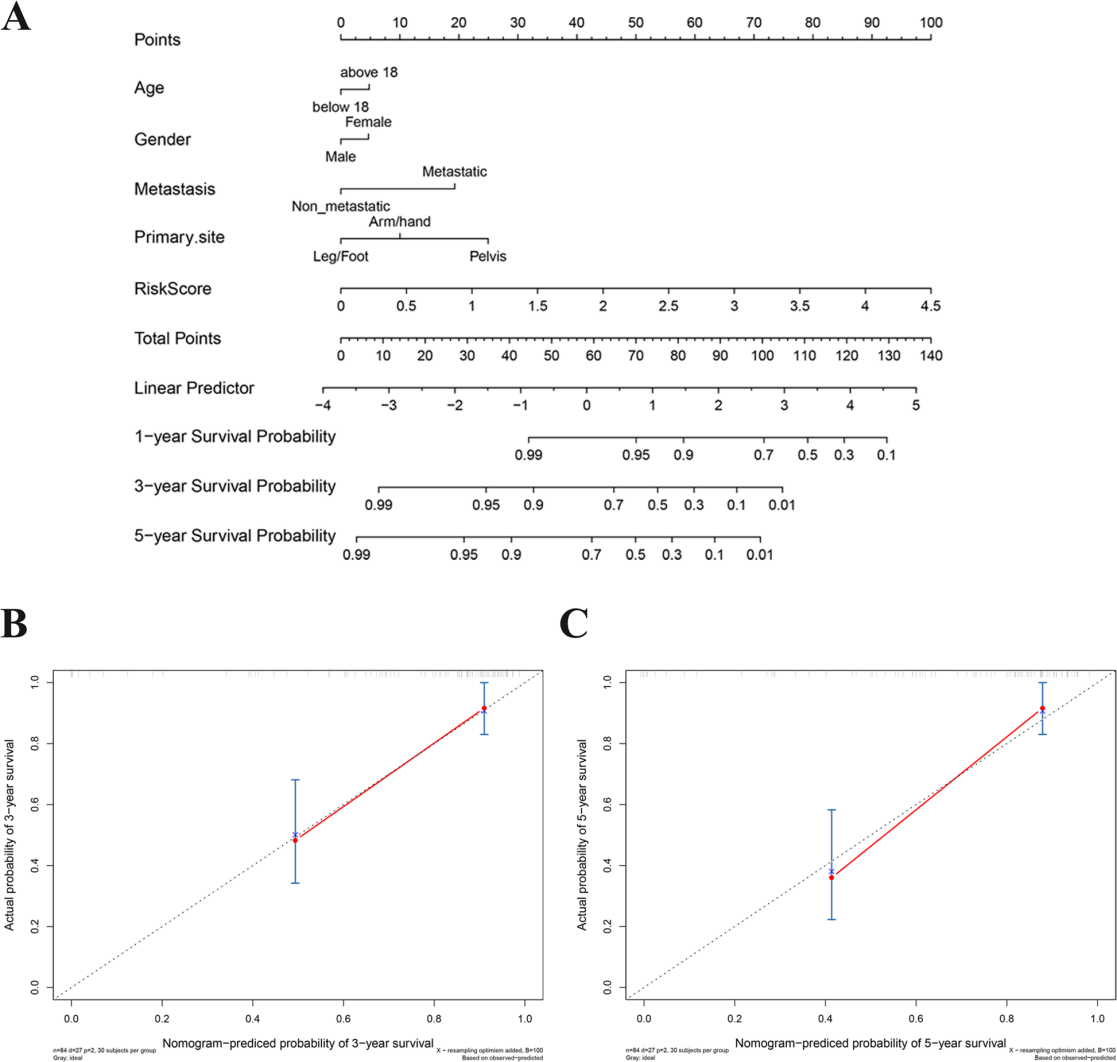
**A** Nomogram model for prognostic prediction of OS patients in the TARGET cohorts. Age, gender, metastasis, primary site, and the risk score were included in the prediction model. **B, C** Calibration plots for prediction of 3- and 5-year survival of OS patients

The event of whether the prognostic value of risk score was influenced by other clinical features was examined using univariate and multivariate Cox regression analysis (Table 2). The risk score was displayed to be independently associated with OS in the TARGET cohorts. Based on these findings, a clinical prognostic nomogram for quantitative prediction of individual overall survival was developed. Age, gender, metastasis, primary site, and the risk score were included in the final OS prediction model (Fig. 8A). The calibration plots in Fig. 8B-C demonstrated that predicted 3-, 5-year OS rates were consistent with the actual observations in the TARGET cohorts to a large extent. Combining the above results, the prognostic nomogram for overall survival prediction is reliable and could be applied in OS patients.

**Table 2:** Univariate and multivariate Cox proportional hazards analyses of clinicopathological variables and risk score in the TARGET cohorts

	n (%)	Univariate analysis HR (95% CI)	*P*	Multivariate analysis HR (95% CI)	*P*
age					
< 18	66(78.57%)	1 (Reference)			
> = 18	18(21.43%)	0.94 (0.36–2.5)	0.91		
Gender					
female	37(44.05%)	1 (Reference)			
male	47(55.95%)	0.72 (0.34–1.5)	0.39		
Primary.Site					
Pelvis	2(2.38%)				
Arm/hand	6(7.14%)				
Leg/Foot	76(90.48%)	1.579(0.15–2.70)	0.54		
Metastasis					
Non metastasis	63(75%)	1 (Reference)			
Metastasis	21&25%)	4.7 (2.2–10)	7.10E-05	6.8(3.0–15.1)	3.42E-06
RiskScore					
Low	42(50%)	1 (Reference)			
High	42(50%)	14 (4.3–48)	1.40E-05	18.7(5.5–63.5)	2.85E-06

## Discussion

Intratumoral heterogeneity refers to the different characteristic of cells with different molecular signatures or differentiation states in a single tumor [19]. At present, intratumoral heterogeneity plays an increasingly important role in tumor treatment, and there is an urgent need to explore cell heterogeneity in osteosarcoma (OS) and related molecular markers using new techniques [20]. Currently, a few studies have explored the osteoclast differentiation in OS, osteoporosis, rheumatoid arthritis, and other diseases by experiments [21–23]. However, cell or animal experiments still have some shortcomings. For example, animal models are not able to recapitulate the human physiological or pathological processes and specificity, 80% of new drug candidates fail to proof their efficacy when tested in human [24, 25]. And bone tumors and cancer metastasis mouse models with injection of human cancer cells also cannot mimic the species-specific mechanisms occuring in human diseases [26]. Therefore, new technologies are needed to conduct research with higher specificity and accuracy.

In present study, we determined 17 separate clusters in primary tumor cells, and 13 separate clusters in metastasis tumor cells by scRNA-seq. Primary and metastasis tumor cells were annotated as different cells. According to the results of cluster annotation of cells, we found that fibroblasts, myeloid cells, osteoblasts, osteoclasts, endothelial cells and proliferating cells were present in both primary and metastatic tumors. The difference is that mesenchymal stem cells and B cells were annotated in metastatic tumors but not in primary tumors, pericytes and T cells were annotated in primary tumors but not in metastatic tumors. For instance, mesenchymal stem cells is the major component of the tumor microenvironmen (TME) [27], Bone marrow mesenchymal stem cells (BMSCs) are mesenchymal stem cells isolated from bone marrow. BMSCs are one of the major components in the TME of OS and are corroborated to mediate proliferation and metastasis of tumor cells [28–30]. B cell responses appear to play an important role in the antitumor immune response in several human tumor types [31]. While their evaluation in sarcomas, including OS, however, has been limited. In this study, we identified significantly elevated B cell infiltrates in metastatic lesions compared to primary canine OS [32]. These results suggest that cell types in the TME change during metastasis of OS. Then, trajectory analysis was applied to split osteoclasts from primary and metastasis tumors into two distinct differentiation state subsets. The subset-dependent ODRGs were identified and GO and KEGG analysis were performed. We found that the functions and pathways of enrichment were different among different differentiation modes.

We used the univariate and Cox-LASSO regression analysis with a process of selection to identify 11 significant survival-predicting ODRGs. Next, the ODRGs-based prognostic risk score model was developed, and its predictive value of prognosis was validated. Combined with the results of Kaplan–Meier analysis, we found that high-risk groups were obviously correlated with shorter survival times. These results indicated that the prognostic risk score model based on ORDGs could be used for patient survival prediction.

Some genes in the 11 ODRGs have been found to function as prognostic biomarkers in other cancers. For instance, increased expression of S100A13 was strongly associated with worse survival in gastric cancer (GC) patients [33]. In addition, compared to para-cancer tissues, S100A13 was expressed at higher levels in hepatocellular carcinoma (HCC) tissues, and higher mRNA expressions of S100A13 was shown to have shorter overall survival. S100A13 may be regarded as a novel prognostic marker in HCC [34]. Tropomyosin alpha-1 chain (TPM1), and proteasome 26S subunit non-ATPase 10 (PSMD10) were the novel predictive biomarkers of GC prognosis [35]. Serine protease inhibitor E2 (SerpinE2), a poor prognostic biomarker of endometrial cancer (EC), promotes the proliferation and mobility of EC cells [36]. Decorin (DCN) was proved to be a promising predictive biomarker for the occurrence and prognosis of lung adenocarcinoma by bioinformatics analyses and experiments [37]. Therefore, combined with the past research literature, the 11 ODRGs are reasonably believed to be a clinical prognostic biomarker.

As a multivariable regression model, the nomogram is applied for predicting clinical outcomes through intuitive visual presentations, and has been widely used in various studies [38]. In our study, we developed a nomogram for predicting OS patient outcomes through ODRGs signature and clinicopathological parameters. Followed, this nomogram has high reliability with TARGET cohort. To date, this nomogram in our study is the first nomogram that predicts OS patient survival with cell differentiation-related signature. Moreover, this study provides a basis for clinicians to predict survival based on clinicopathological and cell differentiation information. However, there are some shortcomings in this study. For instance, the clinical parameters of the patients (medical records, history and tumor imaging results) were incomplete and therefore were not included into the nomogram. In subsequent experiments, it needs to be validated in a large-scale cohort. In addition, this study focused on the use of multiple analytical methods to identify prognostic markers of OS, however, further studies on prognostic markers in animal or cell experiments are needed in the future.

## Supplementary Information

**Additional file 1:** **Table 1. **The canonical markersfor the 10 cell clusters in osteosarcoma tissues.** Table 2. **Eighty Five prognosticassociated ODRGs were selected out by univariate Cox regression analysis in theTARGET OS cohort.** Fig. 1. **Heat map of differentially expressed ODRGs in branches I and IIosteoclasts subsets.

## Data Availability

The datasets used and/or analyzed during the current study are available from the corresponding author on reasonable request.
