# Supplementary material for: Osteoclasts differential-related prognostic biomarker for osteosarcoma based on single cell, bulk cell and gene expression datasets
Source: BMC Cancer. 2022 Mar 17;22:288. doi: 10.1186/s12885-022-09380-z (PMC8932072; doi:10.1186/s12885-022-09380-z)
Supplement: Supplementary file 1 — Additional file 1: Table 1. The canonical markersfor the 10 cell clusters in osteosarcoma tissues. Table 2. Eighty Five prognosticassociated ODRGs were selected out by univariate Cox regression analysis in theTARGET OS cohort. Fig. 1. Heat map of differentially expressed ODRGs in branches I and IIosteoclasts subsets. [file 12885_2022_9380_MOESM1_ESM.docx]

**Table 1. The canonical markers for the 10 cell clusters in osteosarcoma tissues.**

| Cell cluster | Marker genes | Supplementary References |
| --- | --- | --- |
| Osteoblastic cells | Runx2, Col1a1, Cdh11, Ibsp | ^1^ |
| Osteoclasts | ACP5, Ctsk, Mmp9 | ^2^ |
| Myeloid cells | Cd74, Cd14, Fcgr3a | ^3,4^ |
| T cells | CD3, IL7R, CD8a, CD4, Nkg7 | ^5^ |
| Fibroblasts | Dcn, Col1a1 | ^5^ |
| Pericytes | Rgs5, Acta2 | ^5^ |
| Mesenchymal stem cells | Mme, Thy1, Cxcl12, Sfrp2 | ^1^ |
| Endothelial cells | Pecam1, Vwf | ^5^ |
| B cells | Ms4a1, Cd19, Jchain | ^5^ |
| Proliferating cells | Mki67, Top2a, Pcna | ^5^ |

**Table 2. 85 prognostic associated ODRGs were selected out by univariate Cox regression analysis in the TARGET OS cohort.**

| Genes | β-value | HR (95% CI for HR) | *P* |
| --- | --- | --- | --- |
| APOE | -0.25 | 0.78 (0.61-0.99) | 0.043 |
| NPC2 | -0.44 | 0.65 (0.45-0.92) | 0.017 |
| GRN | -0.57 | 0.56 (0.37-0.85) | 0.0064 |
| FCER1G | -0.29 | 0.75 (0.58-0.97) | 0.026 |
| FOLR2 | -0.27 | 0.77 (0.61-0.96) | 0.021 |
| VAMP8 | -0.29 | 0.75 (0.57-0.99) | 0.041 |
| GYPC | -0.31 | 0.73 (0.54-1) | 0.047 |
| MS4A4A | -0.31 | 0.74 (0.56-0.96) | 0.026 |
| SDCBP | -0.63 | 0.53 (0.3-0.95) | 0.032 |
| BRI3 | -0.58 | 0.56 (0.31-1) | 0.048 |
| INTS6 | 0.86 | 2.4 (1.2-4.6) | 0.011 |
| ID2 | 0.52 | 1.7 (1-2.7) | 0.038 |
| CCL2 | -0.35 | 0.71 (0.55-0.91) | 0.0069 |
| TPM1 | -0.67 | 0.51 (0.33-0.79) | 0.0026 |
| BEX3 | 0.47 | 1.6 (1-2.5) | 0.031 |
| SERPINH1 | 0.72 | 2 (1.2-3.5) | 0.0082 |
| PTN | -0.2 | 0.82 (0.7-0.96) | 0.015 |
| COL5A2 | 0.68 | 2 (1.3-3) | 0.0018 |
| MDK | -0.37 | 0.69 (0.5-0.95) | 0.021 |
| SERPINE2 | 0.49 | 1.6 (1.2-2.2) | 0.00098 |
| TUBA1A | -0.81 | 0.44 (0.26-0.75) | 0.0023 |
| CALD1 | -0.42 | 0.66 (0.47-0.92) | 0.015 |
| DCN | -0.37 | 0.69 (0.53-0.89) | 0.0043 |
| S100A13 | 0.78 | 2.2 (1.3-3.5) | 0.0014 |
| OLFML3 | -0.29 | 0.75 (0.57-0.99) | 0.039 |
| CPE | 0.44 | 1.6 (1.2-2) | 0.0011 |
| IFITM3 | -0.46 | 0.63 (0.42-0.95) | 0.029 |
| MYL9 | -0.36 | 0.7 (0.51-0.95) | 0.022 |
| RPL10 | 0.53 | 1.7 (1-2.8) | 0.042 |
| CNN3 | -0.43 | 0.65 (0.43-0.99) | 0.043 |
| ST3GAL4 | 0.98 | 2.7 (1.4-4.9) | 0.0018 |
| GPX7 | 0.77 | 2.2 (1.2-3.8) | 0.0093 |
| IL13RA2 | -0.29 | 0.75 (0.6-0.94) | 0.013 |
| FBLN1 | -0.18 | 0.83 (0.7-0.99) | 0.038 |
| CD320 | 0.5 | 1.7 (1.1-2.5) | 0.017 |
| PANX3 | 0.19 | 1.2 (1-1.4) | 0.023 |
| FDX1 | 0.95 | 2.6 (1.5-4.6) | 0.001 |
| EFNA1 | 0.48 | 1.6 (1.1-2.4) | 0.014 |
| HSPB11 | 0.77 | 2.2 (1.2-3.8) | 0.0072 |
| NREP | -0.69 | 0.5 (0.31-0.82) | 0.0064 |
| CRYBA2 | 0.17 | 1.2 (1-1.4) | 0.034 |
| LOXL1 | -0.28 | 0.75 (0.62-0.92) | 0.0055 |
| MINDY2 | 0.87 | 2.4 (1.4-4.1) | 0.0016 |
| TCEA3 | 0.6 | 1.8 (1.2-2.8) | 0.0042 |
| TMEM119 | 0.45 | 1.6 (1.1-2.3) | 0.02 |
| MT1G | 0.22 | 1.2 (1-1.5) | 0.041 |
| ISLR | -0.12 | 0.89 (0.79-1) | 0.045 |
| DCUN1D5 | 0.43 | 1.5 (1.1-2.1) | 0.0059 |
| CADM1 | 0.48 | 1.6 (1.1-2.5) | 0.027 |
| RPL35A | 0.63 | 1.9 (1.1-3.3) | 0.027 |
| TUBB | -1.1 | 0.33 (0.16-0.7) | 0.0037 |
| PEF1 | -1.1 | 0.35 (0.16-0.77) | 0.009 |
| EI24 | 0.78 | 2.2 (1.1-4.3) | 0.026 |
| MGST1 | -0.16 | 0.85 (0.73-1) | 0.045 |
| RPL37A | 0.55 | 1.7 (1.2-2.6) | 0.0053 |
| G6PD | -0.91 | 0.4 (0.22-0.73) | 0.0027 |
| PSMD10 | -1.2 | 0.3 (0.14-0.64) | 0.0017 |
| PRKRA | 0.77 | 2.2 (1.1-4.3) | 0.029 |
| NUDT1 | 0.44 | 1.6 (1-2.4) | 0.042 |
| CENPW | -0.52 | 0.6 (0.36-1) | 0.049 |
| RPS25 | 0.47 | 1.6 (1.1-2.3) | 0.0065 |
| RPS27 | 0.7 | 2 (1.2-3.3) | 0.0046 |
| ATP2B1 | 0.57 | 1.8 (1-3.1) | 0.045 |
| GADD45GIP1 | 0.47 | 1.6 (1-2.5) | 0.039 |
| PDZD11 | -0.83 | 0.44 (0.22-0.86) | 0.016 |
| FAM207A | 0.74 | 2.1 (1.3-3.5) | 0.0038 |
| RPL13A | 0.64 | 1.9 (1.1-3.4) | 0.027 |
| C19orf48 | 0.5 | 1.6 (1-2.7) | 0.049 |
| PMEPA1 | -0.44 | 0.64 (0.47-0.89) | 0.0074 |
| EBPL | 0.58 | 1.8 (1.1-2.9) | 0.023 |
| PSMB2 | -0.95 | 0.39 (0.16-0.92) | 0.032 |
| PTS | 0.83 | 2.3 (1.2-4.4) | 0.013 |
| ZPR1 | 0.71 | 2 (1.1-3.6) | 0.016 |
| NOP58 | 0.96 | 2.6 (1.3-5.2) | 0.0066 |
| RPS27A | 0.59 | 1.8 (1.1-3) | 0.021 |
| TMEM126B | 0.62 | 1.9 (1-3.3) | 0.039 |
| IER2 | 0.66 | 1.9 (1.1-3.3) | 0.015 |
| NBDY | -0.54 | 0.59 (0.37-0.93) | 0.023 |
| TXNL4A | 0.79 | 2.2 (1.2-4) | 0.0098 |
| EEF1D | 0.88 | 2.4 (1.3-4.4) | 0.0048 |
| PTMA | 0.71 | 2 (1.1-3.7) | 0.019 |
| RPL36 | 0.46 | 1.6 (1.1-2.3) | 0.022 |
| RPL17 | 0.46 | 1.6 (1-2.5) | 0.047 |
| GADD45B | 0.47 | 1.6 (1.1-2.3) | 0.014 |
| TAGLN | -0.44 | 0.64 (0.47-0.88) | 0.0066 |

**Fig. 1.** Heat map of differentially expressed ODRGs in branches I and II osteoclasts subsets.


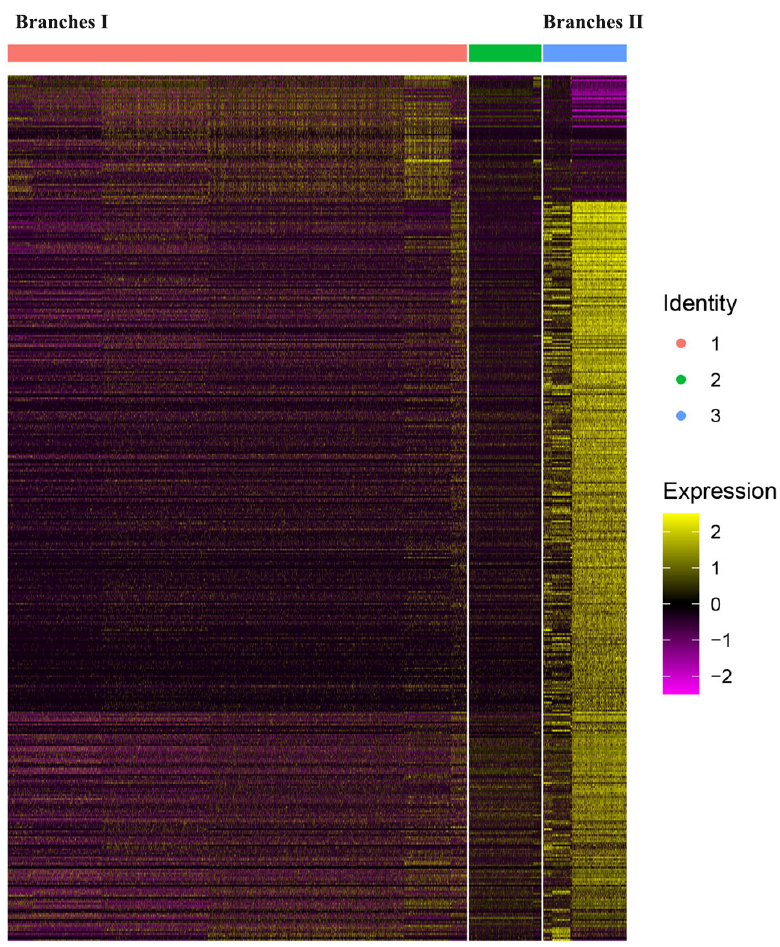


**References**

1 Baryawno, N. *et al.* A Cellular Taxonomy of the Bone Marrow Stroma in Homeostasis and Leukemia. *Cell* **177**, 1915-1932.e1916, doi:10.1016/j.cell.2019.04.040 (2019).

2 Aliprantis, A. O. *et al.* NFATc1 in mice represses osteoprotegerin during osteoclastogenesis and dissociates systemic osteopenia from inflammation in cherubism. *The Journal of clinical investigation* **118**, 3775-3789, doi:10.1172/jci35711 (2008).

3 Guo, X. *et al.* Global characterization of T cells in non-small-cell lung cancer by single-cell sequencing. *Nature medicine* **24**, 978-985, doi:10.1038/s41591-018-0045-3 (2018).

4 Zhang, Q. *et al.* Landscape and Dynamics of Single Immune Cells in Hepatocellular Carcinoma. *Cell* **179**, 829-845.e820, doi:10.1016/j.cell.2019.10.003 (2019).

5 Kim, N. *et al.* Single-cell RNA sequencing demonstrates the molecular and cellular reprogramming of metastatic lung adenocarcinoma. *Nature communications* **11**, 2285, doi:10.1038/s41467-020-16164-1 (2020).
